# Treatment of Pneumonia

**Published:** 1843-08

**Authors:** 


*Treatment of Pneumonia.*—Some very interesting results have been obtained by Grisolle in reference to the influence of blood-letting on the progress of pneumonia from the first to the second stage; and as he advises blood-letting much in the same way as we do in this country, I may shortly notice his observations in order to contrast them with what he found to occur in cases treated by antimony alone. He bled forty-six cases in the first stage of the disease, and yet all, with the exception of ten, passed into the second. He treated forty-four cases with antimony alone, and they were not selected because improper for blood-letting, or because the disease was trivial, for in thirty-five it had already passed into the second stage, and one in seven of the whole number died. The effects of the antimony were much more strikingly beneficial than those of the blood-letting had been in the other cases. In those who recovered, the principal *symptoms* began to amend on the first or second day for the most part, and the local signs continued to increase for several days only in a single instance, while in eighteen cases they began to improve at the end of the first day. That blood-letting should have been practised in some of these cases he admits, referring more especially to three of the six cases that died, in which he says that the circulation was unusually vigorous. We learn from these various researches, not that the one or other practice should be exclusively adopted, but that it is not necessary to repeat the blood-letting so often as the patient seems capable of bearing it—that much of the cure may be safely entrusted, in a great many cases that would still admit of depletion, to the antimony—and that, since antimony alone is evidently so potent a remedy, we have no reason to despair of ultimate recovery in cases that will not bear the evacuation of blood—a remark which can seldom be made of any other considerable inflammatory disease than pneumonia.—*Prof. Henderson, Lond. and Edin. Monthly Journal. Medical Examiner.*

